# Feasibility of a Home-Based Task-Switching Training in Middle-Aged Caregivers

**DOI:** 10.1007/s41465-021-00237-0

**Published:** 2022-01-07

**Authors:** Sarah Susanne Lütke Lanfer, Sören Enge, Marlen Melzer, Jürgen Wegge, Matthias Kliegel

**Affiliations:** 1 Department of Psychology, University of Geneva, Geneva, Switzerland; 2 Clinic of Psychosomatic Medicine and Psychotherapy, University Medical Center Freiburg, Freiburg, Germany; 3 Department of Psychology, Medical School Berlin, Berlin, Germany; 4 Federal Institute for Occupational Safety and Health, Regional Transfer/Special Sectors, Dresden, Germany; 5 Department of Psychology, TU Dresden, Dresden, Germany; 6 Centre for the Interdisciplinary Study of Gerontology and Vulnerability, University of Geneva, Geneva, Switzerland

**Keywords:** Executive control, Task-switching, Real life, Middle age, Training, Adherence

## Abstract

The current study aimed at investigating feasibility of a self-administered task-switching training in a middle-aged working population. Eighty-one caregivers (41–62 years old) were instructed to train at home 8 times either within a 7- or 14-day interval. Only 56.7% performed more than 50% of the instructed number of training sessions. However, compliant caregivers (who completed more than 4 training sessions) showed significant training gains and transfer to an untrained task-switching task. Although transfer effects to other cognitive tasks were not found, trained participants tended to report fewer everyday memory failures than a control group. In conclusion, the implementation of a home-based task-switching training in everyday life of caregivers is possible. However, there is only limited evidence for generalization of results of previous laboratory studies. Adherence and transfer to other cognitive tasks are discussed as important challenges in conveying laboratory findings into real life.

## Introduction

Cognitive plasticity, defined as the potential to change/improve one’s cognitive abilities and brain functions (Baltes, 1987), has been a popular research field, particularly in the last decade. An increasing number of studies that investigate the possibility of training-induced cognitive plasticity have been published. In general, there are two suggested approaches to enhance cognitive performance by training (Morrison & Chein, 2011): First, *strategy-based training*, which focuses on enhancing domain-specific functions, such as encoding or retrieval, in a compensatory way (e.g., if–then plans, imagery, method of loci; e.g., Verhaeghen et al., 1992). Second, *process-based training*, which aims at improving a particular cognitive ability (e.g., working memory) by repetitively exercising the underlying domain-general core mechanisms (e.g., executive control, processing speed; e.g., Borella et al., 2010). Findings in both training areas suggest that cognitive plasticity in trained abilities may be possible in various populations (see meta-analyses: old age, e.g., Hudes et al., 2019; Karbach & Verhaeghen, 2014; Karr et al., 2014; Kelly et al., 2014; Malmberg Gavelin et al., 2020; Melby-Lervag & Hulme, 2013; Mewborn et al., 2017; Tetlow & Edwards, 2017; cognitive impairment, Bahar-Fuchs et al., 2019; Hill et al., 2017; Li et al., 2004; Reijnders et al., 2013; Teixeira et al., 2012; children, Karch et al., 2013; Luis-Ruiz et al., 2020; Rapport et al., 2013). With regard to process-based training studies, training benefits and transfer to other cognitive tasks in comparison to control groups were more frequently reported than for strategy training studies. However, the pattern remains rather ambiguous, and the precise mechanisms that have led to such performance benefits in some studies are still unclear (for example, it is still under debate whether actual cognitive changes took in fact place or whether alternative explanations such as task-specific learning or motivation effects are more likely candidates explaining the results; Enge et al., 2014; Melby-Lervåg & Hulme, 2016; Redick et al., 2015; Sala et al., 2019; Shipstead et al., 2010, 2012). Nevertheless, there is no doubt that executive control such as working memory (WM) or task-switching addressed in these studies are key to both complex/higher-order cognitive abilities in the lab and in everyday life (e.g., PM, fluid intelligence) and age-related declines in those domains (e.g., Li et al., 2004; Schnitzspahn et al., 2013). Besides the fundamental concerns, in view of an aging population (and workforce) and the importance of adequate cognitive abilities for job performance and independence in old age (see, e.g., Elvevag et al., 2003; Hering et al., 2018; Ihle et al., 2015; Kliegel et al., 2008; Schmidt & Hunter, 1998, 2004), investigating the possibilities and limitations of process-based training regimes in healthy adults, specifically in its relation to application in everyday life, is of particular relevance to applied aging and cognitive research. The present project explicitly targeted these issues and thus represents one of few studies to investigate feasibility of process-based training in everyday life of healthy adults.

### Popularity of Process-Based Training

At the end of the 2000s, the potential of successful process-based training was suggested by a handful of studies promising substantial transfer effects to untrained complex transfer tasks. In particular, studies reporting transfer to fluid intelligence measures caused (possibly unwarranted) optimism, since mixed results from other training studies have implied that fluid intelligence is relatively fixed (e.g., Sternberg, 2008). A very prominent study by Jaeggi and colleagues (2008) found that due to an adaptive dual *n*-back training over a period of 8 to 19 days, 34 healthy young participants improved in the training task and when compared to a passive control group (*N* = 35) in a measurement of fluid intelligence (here: reasoning task). Jaeggi and colleagues (2010) confirmed and extended their findings in another study, showing performance improvement to two intelligence measures for the dual and single n-back training groups compared to a passive control group. While some commentaries underlined the importance of the work of Jaeggi and colleagues (e.g., Sternberg, 2008; S. Wang & Aamodt, 2009), Moody (2009) criticized that firm conclusions for actual changes in fluid intelligence ability cannot be drawn as several critical parameters such as motivational effects were not adequately controlled (e.g., Shipstead et al., 2010, 2012).

After the training of task-switching, another important basic process, Karbach and Kray (2009) found substantial training gains and transfer effects in three different age groups (8–10; 18–26; 62–76 years of age) after 4 weekly training sessions (30–40 min) compared to an active control group. For all age groups, performance improvements in a structurally similar switching task, in inhibition, verbal and spatial work memory and also in a fluid intelligence measure were revealed. Other studies which used the same self-paced task-switching paradigm found similar results within different age groups and reported training and transfer effects to, for example, untrained WM tasks (e.g., adolescence, Zinke et al., 2012). Moreover, recent MRI studies showed even neuronal changes in older participants after the same task-switching training (Dörrenbächer et al., 2019, 2020).

Since these first studies, several reviews and meta-analyses underlined the possibility that training of executive control tasks (e.g., WM updating, task-switching) may lead to enhancement of performance in other untrained cognitive tasks (e.g., Au et al., 2016; Karbach & Verhaeghen, 2014; Malmberg Gavelin et al., 2020; Mewborn et al., 2017). Yet, some authors argued that although these studies found transfer effects, overall process-based training studies show inconsistent and elusive results (e.g., Melby-Lervag & Hulme, 2013; Melby-Lervåg & Hulme, 2016; Redick et al., 2013; Sala et al., 2019; Shipstead et al., 2012). However, there is a great variety of training and transfer tasks applied in these studies. As pointed out by Morrison and Chein (2011) and recently by Smid et al. (2020), differing training techniques and regimes seem to produce different impacts on untrained cognitive abilities. In their systematic review, for example, Mewborn et al. (2017) found dissimilar effect sizes for different one-domain process-based trainings. In a systematic overview over systematic reviews, Malmberg Gavelin et al. (2020) detected an overall effect of cognitive-based training interventions. However, the great heterogeneity of studies and the lack of high-quality review studies made interpretation of results difficult. Thus, to resolve the debate about the effectiveness of process-based training, more detailed approaches in meta-analytic reviews as well as more comparable research ought to be necessary. In regard to far transfer, particularly the impact of processed-based training on everyday functioning is largely under-investigated (e.g., Kelly et al., 2014; Malmberg Gavelin et al., 2020; Mewborn et al., 2017; Reijnders et al., 2013).

### Process-Based Training and Everyday Life

From an applied perspective, cognitive training research targets the question of applying lab-based findings to real-life scenarios and daily activities (Schubert et al., 2014). To date, however, such applied process-based training studies examining training outside the laboratory are very limited. Targets have predominantly been groups with supposed limited cognitive capacity, such as children, older adults, or participants with impairments/performance deficits (e.g., Anguera et al., 2013; Bergman-Nutley & Klingberg, 2014; Titz & Karbach, 2014). However, as executive control and/or fluid cognitive processes performance are assumed to peak between the age of 20 and 25 and to have linear age-related decline thereafter (e.g., Schaie, 1994), healthy middle-aged adults are a key and so far neglected target group. Importantly, following up on the predictions of a disuse theory of adult cognitive decline, strengthening cognitive processes could prevent early decline (e.g., Ihle et al., 2020a, 2020b, 2020c; Stern, 2009; Wilson et al., 2013) and buffer decline/impairment through stress and help dealing with work demands (e.g., multi-tasking, interruptions, inhibition of undesired emotions; Gajewski et al., 2010; Gajewski et al., 2020; Ihle et al., 2015; Loft et al., 2019). Moreover, due to the usual channels of recruitment in most experimental psychological studies, so far, limited evidence exists on non-university populations. One applied training study targeting middle-aged adults introduced a training regime comprising several tasks (e.g., speed tasks, span tasks, memory tasks, Sudoku; Mental Activation Training MAT, Lehrl et al., 1994) into the daily routine of car-factory workers (Gajewski et al., 2017). Participants trained twice a week for 3 months which led to significant improvement in task-switching. In addition, authors reported changes on brain function 3 months after training. Furthermore, small improvements between pre- and post-test results were reported for an attention and a fluid intelligence measure (Freude et al., 2011). As this example shows, in contrast to the laboratory trainings described above, cognitive trainings implemented in daily routines are mostly multi-domain interventions (e.g., video game training, P. Wang et al., 2016; tablet training, Chan et al., 2016). However, the complex nature of a multi-domain training makes it difficult to determine which specific feature or process of the training regime induces transfer (cf. e.g., Karbach & Verhaeghen, 2014). In contrast, with a single-domain process-based training, the targeted cognitive domain (e.g., task-switching) can be explicitly chosen and aligned with specific (work) demands. Although laboratory evidence suggests that some one-domain process-based trainings may be effective, to our knowledge, no study has examined implementation into daily routine of (healthy) working adults.

### The Present Study

The central aim of the present project was to investigate implementation of a successful laboratory training regime in a less controlled everyday life environment of healthy working adults. Thus, we conducted a feasibility study of applying an established one-domain process-based laboratory training in daily life of middle-aged caregivers. To answer the feasibility question, we targeted four areas: *adherence* (do working adults perform home-based process training as instructed without outside assistance), *effectiveness* (do healthy adults benefit from performing process-based training in everyday life), *efficiency* (what is the most efficient, less time-consuming way to benefit from process-based training in everyday life), and *generalization* (do laboratory results generalize to a less controlled and monitored everyday life environment).

For the present study, we intentionally chose a non-academic working population (workers in the elderly care field) that should especially benefit from strengthened cognitive resources to cope with intense and changing work demands. Thus, as enhanced cognitive resources can have a strong and beneficial long-term effect on health and work ability, *adherence* to cognitive training is of particular interest in such a target group. Matching the work specific demands in nursing with laboratory process-based training results, we modeled our training regime closely after the study by Karbach and Kray (2009). Several reasons contributed to our decision: First, their task-switching training showed training gains and transfer effects to other cognitive tasks that could be replicated in different studies (see above).

Second, training effects were found for several age groups (children, adolescents, students, and older adults), which points towards *generalization* of training effects in the laboratory. Third, from our previous work, there is evidence from a theoretical and empirical perspective that cognitive flexibility is important in handling common nursing-related work demands (e.g., multitasking, dealing with undesired emotions) and seems to be a predictor for work ability in nursing (e.g., Ihle et al., 2015; Loft et al., 2019).

In line with current training research, to determine *effectiveness*, we explored (1) whether the task-switching training produces training gains when performed self-dependent outside the laboratory and (2) whether the training induces transfer to a structurally similar but untrained task (near transfer), (3) to tasks measuring other cognitive domains (mid or far transfer), or (4) changes in everyday life (self-observed transfer). In 4, a cued task-switching task, an updating task, an inhibition task, and two prospective memory tasks (laboratory vs. self-report) were chosen to investigate transfer. Considering Miyake et al. (2000), we chose updating and inhibition as possible transfer domains as these two functions are clearly separable from task-switching but still core executive processes. Moreover, both applied tasks are well-researched, popular, and have been used as transfer tasks for task-switching training before (e.g., Karbach & Kray, 2009; Zinke et al., 2012). Theoretically, task-switching training should not lead to large transfer to updating or inhibition. However, previous studies found transfer to inhibition (Karbach & Kray, 2009) and updating (Zinke et al., 2012) for a similar task-switching training. The rationale behind prospective memory as transfer task lies in the theoretical and empirical established relevance of executive control (particularly, task-switching) for prospective memory and its age-related decline (see, e.g., Gray-Burrows et al., 2019; Schnitzspahn et al., 2013; Zuber et al., 2016). Hypothetically, strengthening a key underlying mechanism (i.e., task-switching) should lead to better prospective memory performance and buffer decline of prospective memory with advancing age.

Lastly, there is only a handful of research investigating the role of spacing (time between training sessions) on training effectiveness (e.g., see Jaeggi et al., 2020; Z. Wang et al., 2014), which mostly investigates very short intervals between sessions (e.g., twice per day, once per day, or once every-other-day, see Jaeggi et al., 2020). However, this seems very time-consuming for working participants and an increased hurdle to start cognitive training. Thus, we further aimed to identify the most *efficient* and less time-consuming training interval between sessions. Therefore, we manipulated training intervals and instructed participants to either train one session every week according to Karbach and Kray (2009; 7-day interval) or one session every fortnight (14-day interval).

Taken together, the present study investigated the four feasibility questions, (1) whether caregivers perform task-switching training self-dependent in their daily life (*adherence*), (2) whether the task-switching training produces training and transfer gains when performed self-dependent outside the laboratory (*effectiveness*), (3) whether a weekly (7-day) or a fortnightly (14-day) interval between training sessions is most beneficial for caregivers (*efficiency)*, and (4) whether previous laboratory results for adults (e.g., Karbach & Kray, 2009) generalize to the everyday life of middle-aged caregivers (*generalization*). To our knowledge, the present study is the first to address these issues.

## Method

### Participants and Design

**Fig. 1:**
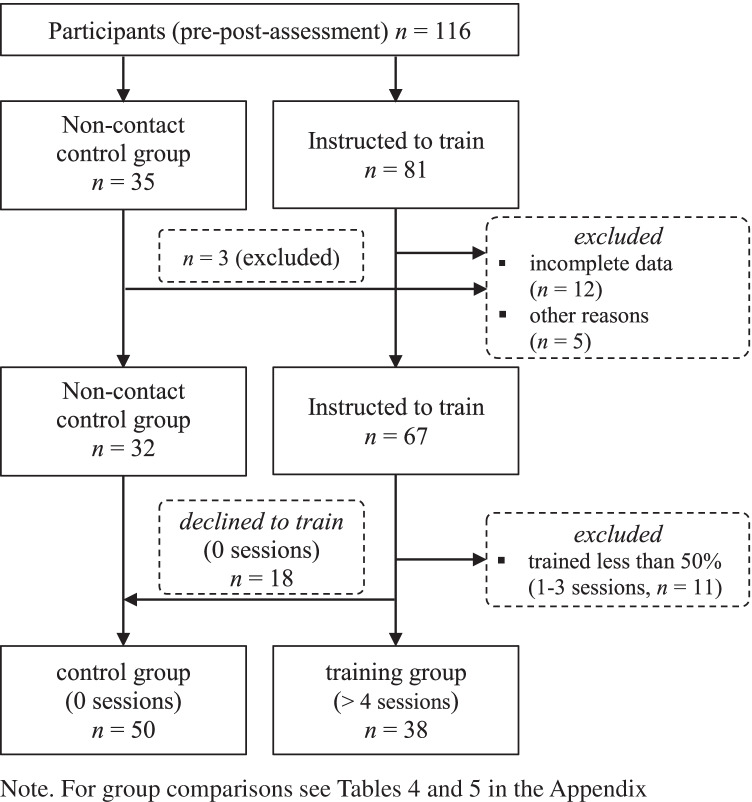
Flow diagram of group compositions

The primary sample consisted of 116 caregivers who were working in 16 nursing homes in Germany. Data was collected within a larger field study focusing on strengthening subjective health and work ability in middle-aged care workers. For the purpose of this study, we only analyze data of care workers. Mean age was 52.68 years (*SD* = 5.00 years), ranging from 41 to 62 years. Eighty-one participants were instructed to perform a task-switching training at home, while 35 nurses did not receive any instruction for cognitive training at home. Both groups did not significantly differ in regard to age, gender, average working hours, and pre-test task-switching performance. Participants did not report any neurological/ psychological disease or color blindness. Seventeen participants had to be excluded from the sample due to incomplete or missing data (*n* = 12), lack of language skills (*n* = 3), and outside assistance during training sessions at home (*n* = 2). Thus, the *final study sample* comprised 99 elderly care workers (mean age = 52.59, *SD* = 5.18, 3 male), of which 67 were instructed to train at home (see Fig. 1). Excluded participants did not significantly differ concerning age, gender, average working hours, and pre-test task-switching performance (Table 4 in the Appendix).

The study followed a three factorial design with two between-subject factors, *cognitive training* (yes/no) and instructed time *interval* between training sessions (7 days/14 days), and one within-subject factor, *time* of assessment (pre-training/post-training). Nursing homes were randomly assigned to the two different interval groups (7 days vs. 14 days interval), and participants in each institution then were allocated to training and control groups, respectively. Thus, participants were pseudo-randomly assigned to one of four groups: two cognitive training groups, one with a shorter between training interval (7-day training group) and one with a longer between training interval (14-day training group); and for each training interval condition, a control group, that did not complete any cognitive training session at home but for which the pre-post interval matched (7-day control group and 14-day control group, respectively). Participants did not receive any monetary *gratification*; however, most retirement homes granted the pre- and post-assessment hours (2–3 h) as advanced vocational training time. After completing the project, all participants received a certificate and information about the anti-stress and selection-optimization-compensation training and had the opportunity to obtain individual performance feedback.

### Materials and Tasks

#### Task-Switching Training

For the training of executive control, a task-switching paradigm was used (inspired by Karbach & Kray, 2009). The training comprised 24 blocks á 17 trials, which means a total of 408 stimuli were presented. Participants were given two simple decision tasks (tasks A and B) and were instructed to switch tasks on every second trial. Task A (“letter” task) required participants to decide whether the presented stimuli was an upper- or lower-case letter and task B (“direction” task) whether the letter was presented vertically or horizontally. The stimuli set only comprised letters that figuratively differ as an upper- and lower-case letter; each upper- and lower-case letter was presented in a vertical (standard) or horizontal position. Each target presented was introduced by a fixation cross (1400 ms) and followed by a 25 ms after response delay. The target was presented until participants indicated their answer by pressing a response key (left and right arrow key, respectively). The training program was stored on a USB stick and could be used on any Microsoft Windows-based personal computer. Letters were always presented in the middle of the screen in a fixed size (front: Arial 200). Participants were instructed to respond as fast and as accurately as possible. After each block performance feedback (correct responses, average time per trial) was given. Training sessions lasted about 20 min. Main dependent variables were switching costs (difference in mean performance between switch and non-switch trials), reaction time (RT), and accuracy (see, e.g., Kray & Lindenberger, 2000).^1^

#### Transfer Tasks (Pre- and Post-training Assessments)

In a pre-post-test design, it was assessed whether the home-based self-administered task-switching training, performed in everyday life, causes transfer to related cognitive domains, using a battery of cognitive tests (administered in a laboratory-type session at work, see below) for which participants were instructed to respond as quickly and as accurate as possible.

**Task-Switching**: To examine near transfer to a structurally dissimilar *task-switching* task, a random number switch task based on an external cueing paradigm was used (for a review on switching paradigms, see, e.g., Kiesel et al., 2010). Next to the number (1–4 and 6–9), the decision task (task A or B) was presented on the computer screen (fixed cue-stimulus interval of 0 ms). Participants were required to decide whether the number presented was smaller or larger than five (task A, “size” task) or odd or even (task B, “odd/even” task). Each target was present until a response was made and followed by a blank inter-stimulus interval of 1500 ms. The assessment consisted of three blocks: two single task blocks (80 trials) and one mixed block (80 trials). Each single task block contained only one task (task A or B), whereas in the mixed block, both tasks (tasks A and B) were randomly intermixed. In about half of the trials participants had to switch between the task (switching trials), and for the remaining trials, they had to repeat the previous task. At the beginning, each block contained practice trials for participant to familiarize themselves with the task (8 trials/single task blocks, 16 trials/mixed task block)^1^. Two types of task-switching costs were analyzed for RT and accuracy: *switching costs* as the difference in mean performance between switch and non-switch trials within mixed blocks and *mixing costs* as the difference in mean performance (errors) between mixed and single task blocks.

**Inhibition:** The Color-Stroop interference task (Stroop, 1935; German version from the Nürnberger-Alters-Inventar, NAI; Oswald & Fleischmann, 1999) was used to measure transfer to *inhibitory control*. The task incorporated three blocks containing 36 target stimuli each. First, participants were presented with color words (red, green, blue, and yellow) printed in black on a sheet of paper, which they had to read out loud. In the second block, colored square patches (red, green, blue, and yellow) had to be named. In the interference block, color words were printed in an incongruent ink color and participants had to name the ink color and inhibit the prominent reaction to read the color word. Overall RT for each block was taken by experimenter using a stopwatch. Interference score was calculated by subtracting overall RT in the color ink naming block from overall RT in the interference block.

**Updating:** For exploring transfer to *updating*, a number-based 2-back task was administered. Participants had to decide whether the presented number was the same as the one next to the last pressing the marked key (“yes” left arrow key, “no” right arrow key). Numbers were presented for 2000 ms, followed by a 1000 ms blank inter-stimulus interval. After a short practice of eight trials, participants performed a block of 82 trials (the first two trials were excluded from the data analyzes because there were no next-to-last numbers for these trails). Twenty trials of the presented numbers were targets (2-back trials). Correct mean RT per trial and total correct responses to targets served as dependent measures. Missed trials were counted as errors in the analyses of accuracy data.

**Prospective Memory Performance:** Two different measures were applied to assess far transfer to *prospective memory performance*: a laboratory task and a self-report questionnaire. For the laboratory assessment, an event-based task (i.e., the execution of the intended action is activated by a specific external cue) in two different versions (pre-/post training) was used. At the beginning of the assessment, participants were instructed to state aloud their year of birth/place of residence unsolicited when they encounter the prospective memory cue later during the testing. The cue was hidden in an instruction for another cognitive task. Task was scored as correctly (score = 1) when the intended action was performed as instructed; otherwise, the score was 0 (Kliegel et al., 2007). For subjective PM performance, 4 items from the Prospective and Retrospective Memory Questionnaire (PRMQ, Smith et al., 2000; German version: Zimprich et al., 2011) were used^2^. After the first assessment, the prospective memory scale was reduced to one item for each dimension: (a) short-term vs. long-term memory and (b) self-cued vs. environmentally cued. High correlation (*r* = 0.85) between the 4-item scale and full scale at pre-training assessment suggests adequate representativeness of the 4-item version. Statements about memory functions were to be answered on a 5-point Likert scale (1 = agree strongly, 5 = disagree strongly). Negative items were inverted. Thus, high values indicate a perceived stronger memory problem.

**Questionnaire Data**^2^: *Subjective health* was assessed using the WHO-5 questionnaire (Brähler et al., 2007) and the emotion exhaustion scale from the Maslach Burnout Inventory (MBI, Schaufeli et al., 1996). For the WHO-5, high values indicate well-being, whereas high values in emotional exhaustion point to a risk for burnout. Demographic variables were obtained as well (see Table 1).

**Table 1:** Participants’ mean scores and standard deviations on demographic and cognitive variables shown separately for training and control groups (cognitive variables assessed at pre-test)

	Training groups		Control groups		F-/ *χ*^*2*^-value
	7-day interval	14-day interval	7-day interval	14-day interval	
*N*	18	20	21	29	n.s.
Time between assessments (in weeks)	10.03 (1.73)	18.75 (2.02)	9.90 (1.81)	17.83 (2.66)	n.s
Number of performed training sessions	10.11 (8.66)	9.10 (8.75)	-	-	n.s
*Demographics*					
Sex (female)	18	20	20	27	2.53
Age (years)	53.22 (5.49)	51.95 (5.49)	52.38 (5.56)	53.31 (4.80)	.34
Education (years)	10.00 (1.03)	10.21 (.63)	9.53 (.87)	10.00 (1.24)	1.46
Weekly working hours	32.19 (4.07)	32.08 (4.58)	32.00 (7.18)	31.41 (5.74)	.09
Tenure	12.12 (6.55)	9.17 (9.17)	18.20 (8.91)	16.31 (8.56)	4.54**
Computer competence (self-report)	5.61 (2.06)	4.75 (2.49)	4.71 (2.49)	5.04 (2.76)	.52
*Health*					
Emotional exhaustion	2.93 (1.20)	3.16 (1.27)	3.17 (1.56)	3.80 (1.73)	1.50
Well-being	3.20 (.86)	2.55 (.98)	2.77 (1.16)	2.63 (1.29)	1.19
*Cognitive variables*					
Switching costs (RT)	215 (214)	243 (287)	236 (287)	351 (283)	1.28
Mixing costs (RT)	906 (283)	960 (441)	794 (467)	1159 (489)	3.05*
Switching costs (error)	1.83 (3.81)	4.90 (7.06)	1.71 (5.51)	3.17 (3.90)	1.66
Mixing costs (error)	− 1.06 (6.69)	1.35 (4.80)	1.95 (7.28)	.72 (4.02)	.99

*Note*. *p < .05

^**^p < .01

### Procedure

All participants completed two assessment sessions (pre-/post-training), conducted individually with a trained psychology experimenter in a separate/quite room at the participants’ workplace. Time between assessments was on average 10 weeks for the 7-day interval groups (training vs. control) and 18 weeks for the 14-day interval groups (training vs. control) (see Table 1). Questionnaire data were obtained in separate sessions before and after the assessments. In both, pre- and post-training assessment, all participants completed a battery of cognitive tasks which were computerized (task-switching, updating) and oral (prospective memory, inhibition), respectively. For all computerized tasks, laptops with 17 inch CRT color monitor were used. The sequence of tasks was held constant. Assessment sessions lasted about 60 min.

In addition, *training groups* learned how to apply the task-switching program at pre-training assessment. The training program was stored on a USB-stick and was programmed to start immediately when inserting the USB-stick into the USB-slot at a windows-based computer. After a demonstration of usage of the USB-stick and the task, participants practiced starting and executing the program until they felt confident to do so. Participants were instructed to train either weekly (7-day training group) or every second week (14-day training group) for at least 8 times until the post-training session took place. It was recommended that participants train regularly at the same week day. To help implementation at home, participants received a take-home instruction sheet that explicitly explained the training task and usage of the training program (via USB-Stick) and instructed them to call the researcher if any problem occurs. At the post-training assessment, participants of the training groups handed in their data (via USB-stick) and answered follow-up questions concerning their training behavior (e.g., problems, amount of training, usability, motivation, relevance). After the post-test session, the experimenter checked whether the data was stored on the USB stick and matched the number of stored sessions to the answer of the participant.

## Results

### Adherence to a Home-Based Task-Switching Training in Middle-Aged Caregivers

**Fig. 2:**
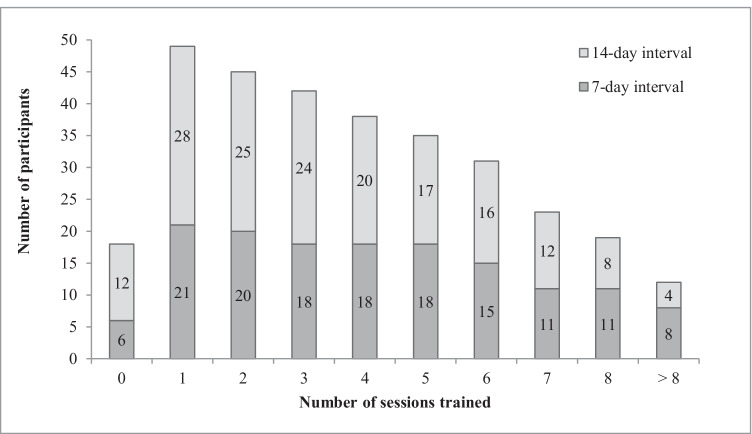
Number of sessions trained as a function of training interval

The distribution of performed cognitive training sessions^3^ at home was as follows: 18 participants trained 0 sessions (26.86%), 11 between 1 and 3 sessions (16.42%), 19 caregivers trained 4 to 7 session (28.36%), and 19 participants 8 or more sessions (28.36%). Only 7 participants conducted 8 training sessions as instructed (10.45%): 3 of the 7-day training group and 4 of the 14-day training group. Twelve nurses trained more than 8 times: 5 performed 9 sessions (7.46%), 5 participants between 10 and 18 times (7.46%), and 2 nurses performed even more than 40^4^ sessions at home (2.99%). The percentage of participants training at least 4 sessions (*N* = 38) was higher for the 7-day training group than for the 14-day training group (66.67% vs. 50.00%; see Fig. 2). Performed training sessions for the eligible participants, who were instructed to train at home, are displayed in Fig. 2.

### Effects of a Home-Based Task-Switching Training in Middle-Aged Caregivers

To explore the feasibility regarding effectivity, efficiency, and generalization, we formed trainings and control group(s) as follows: In line with the training intensity reported by Karbach and Kray (2009), for analyses, only participants training at least 4 times in the respective time interval were included in training group(s) (*N* = 38). The 18 care workers did not train at home (0 sessions) were added to the control group(s) (for comparisons with training and control group, see Table 4 in the Appendix).^5^ Participants training less than 50% of the instructed sessions (1–3 sessions) were excluded from the training groups. Thus, the *final training sample* used to answer the questions regarding effectivity, efficiency, and generalization comprised 88 middle-aged elderly care workers (mean age = 52.86, *SD* = 5.17, 3 male, see Fig. 1). Average number of performed training sessions did not differ significantly between 7-day training group and 14-day training group, *t*(36) = 0.36, *p* = 0.72, *d* = 0.12. Excluded participants (*n* = 11, 1–3 sessions) did not significantly differ in regard to age, gender, average working hours, and pre-test task-switching performance (Table 5 in the Appendix).

#### Training Effects

To investigate training-related benefits, repeated measure ANOVAs with mean RT per trial, accuracy per trail, and switching costs (RT, accuracy) were conducted with session (first vs. fourth/last) as within-subject factor and interval (7-day vs. 14-day) as between-subject factor. First, we compared the first to fourth training (50% of instructed assignment) sessions (mean performance see Table 2). Repeated measure ANOVAs revealed large-sized training effects for mean RT per trial and accuracy, *F*(1, 36) = 43.16, *p* < 0.001, η^*2*^_*p*_= 0.54 and *F*(1, 36) = 16.30, *p* < 0.001, η^*2*^_*p*_= 0.31, respectively. RT switching costs were also reduced from the first to the fourth training session (*F*(1, 36) = 5.35, *p* < 0.05, η^*2*^_*p*_= 0.13). No significant effect was found for switching costs in accuracy, *F*(1, 36) = 0.30, *p* = 0.58, η^*2*^_*p*_= 0.01. For all analyses, no session × interval interaction effects were found (*F*(1, 36) = 0.19–1.75, *p* = 0.19–0.69, η^*2*^_*p*_= 0.01–0.05), indicating that both training groups (7-day vs. 14-day) did show similar training gains over the first four sessions.

**Table 2:** Mean RT and accuracy data for first to fourth and last task-switching training sessions displayed separately for 7-day and 14-day interval training groups

	Training session 1 M (*SD*)	Training session 2 M (*SD*)	Training session 3 M (*SD*)	Training session 4 M (*SD*)	Training last *M* (*SD*)
**7-day interval** (*N* = 18)					
*RT in ms*					
Switch trials	1400 (499)	1127 (362)	1000 (245)	936 (224)	706 (183)
Non-switch trials	1061 (326)	863 (255)	757 (159)	754 (212)	596 (128)
Switching costs	339 (300)	264 (208)	243 (173)	182 (167)	111 (109)
*Accuracy rate in %*					
Switch trials	86.0 (12.2)	91.4 (9.9)	93.4 (7.8)	93.3 (9.0)	93.8 (9.0)
Non-switch trials	87.4 (12.2)	93.0 (9.7)	93.9 (8.1)	93.7 (10.0)	94.3 (10.3)
Switching costs	1.4 (2.57)	1.6 (2.19)	0.6 (1.97)	0.4 (2.61)	0.5 (2.08)
**14-day interval** (*N* = 20)					
*RT in ms*					
Switch trials	1382 (651)	1112 (411)	1038 (435)	946 (392)	871 (343)
Non-switch trials	1178 (510)	1001 (445)	929 (379)	799 (259)	721 (218)
Switching costs	204 (238)	111 (192)	111 (170)	147 (159)	150 (162)
*Accuracy rate in %*					
Switch trials	84.3 (12.3)	86.1 (11.9)	85.9 (12.3)	88.6 (13.0)	91.0 (11.5)
Non-switch trials	85.5 (12.8)	85.9 (13.3)	87.1 (12.0)	90.1 (12.5)	91.5 (13.2)
Switching costs	1.2 (3.37)	-0.3 (2.41)	1.1 (2.46)	1.55 (2.37)	0.4 (2.70)

**Fig. 3:**
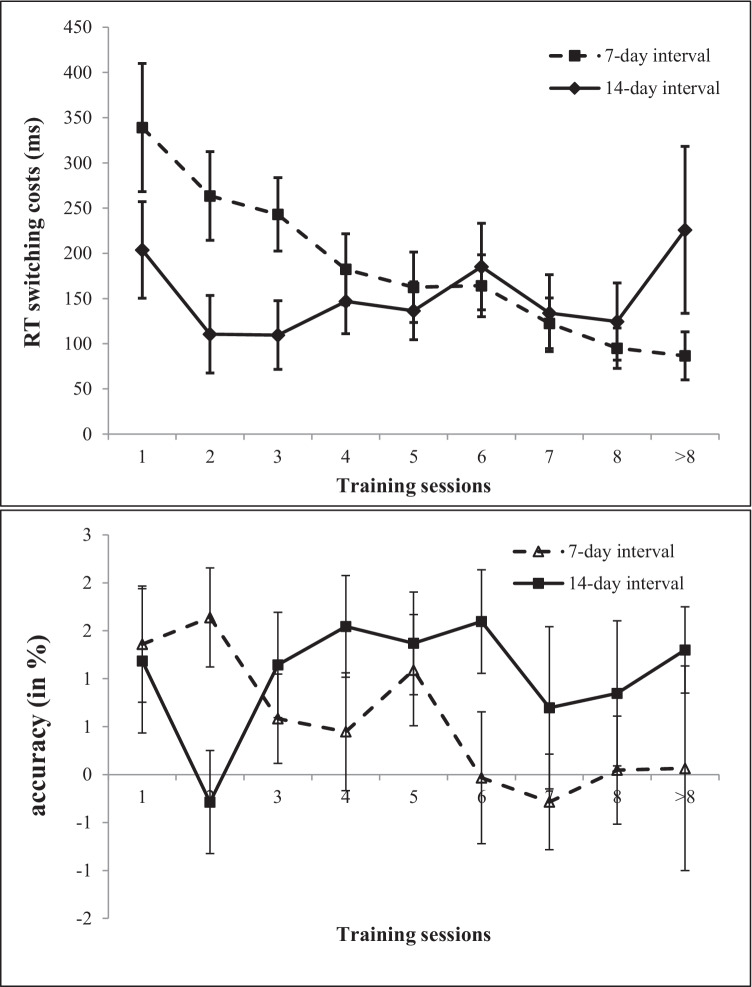
Switching costs (RT, accuracy) for training sessions 1 to 8 for participants in training groups

In a second step, performances in the first and last training sessions were compared (mean perfomance see Table 2).^6^ Results displayed a similar pattern. Significant time effects were found for mean RT per trial and accuracy, *F*(1, 35) = 21.27, *p* < 0.001, η^*2*^_*p*_= 0.38 and *F*(1, 35) = 4.47, *p* < 0.05, η^*2*^_*p*_= 0.11, respectively. Interestingly, for RT switching costs, a main effect for session as well as an interaction effect session × interval was revealed (*F*(1, 35) = 4.42, *p* < 0.05, η^*2*^_*p*_= 0.11 and *F*(1, 35) = 4.33, *p* < 0.05, η^*2*^_*p*_= 0.11, respectively). Participants in the 7-day training group showed greater training gains in RT switching costs than participants in the 14-day training group. Again, for switching costs in accuracy, no significant effect over time was revealed, *F*(1, 35) = 0.02, *p* = 0.89, η^*2*^_*p*_= 0.00. Training gains did not differ for the two interval groups for correct mean RT and both accuracy measures (*F*(1, 35) = 0.03–0.14, *p* = 0.71–0.87, η^*2*^_*p*_= 0.00). Figure 3 shows overall task-switching performance (RT and accuracy) for training sessions 1 to 8 displayed for each training interval group.

#### Transfer Effects to Non-trained Tasks

To assess transfer effects, repeated measure ANOVAs with the between-subject factors group (training/no training) and interval (7-day/14-day) and the within-subject factor time of assessment (pre-training/post-training) were calculated (for mean performance see Table 3). For the external cued *task-switching task*,^7^ no time × group interaction effects were found for RT switching and mixing costs, *F*(1, 84) = 0.13, *p* = 0.72, η^*2*^_*p*_= 0.00 and *F*(1, 84) = 1.40, *p* = 0.24, η^*2*^_*p*_= 0.02, respectively. However, there was a trend towards a time × group × interval interaction effect for RT mixing costs, *F*(1, 84) = 3.75, *p* = 0.056, η^*2*^_*p*_ = 0.04. Post hoc analyses revealed a trend towards a time × group interaction effect (i.e., transfer effect) for the 7-day interval instruction, but not for the 14-day interval (*F*(1, 37) = 2.79, *p* = 0.10, η^*2*^_*p*_ = 0.07 vs. *F*(1, 47) = 0.59, *p* = 0.45, η^*2*^_*p*_ = 0.01). A medium-sized time × group interaction effect was found for accuracy switching costs, *F*(1, 84) = 6.74, *p* < 0.05, η^*2*^_*p*_ = 0.07. Participants who performed task-switching training at home showed a larger decrease in accuracy switching costs than participants in the noncontact control groups. For mixing costs in accuracy, analysis found no time × group interaction effect (*F*(1, 84) = 0.20, *p* = 0.65, η^*2*^_*p*_ = 0.00).

**Table 3:** Mean performance (SD) on task-switching task and far transfer variables as a function of session (pre-test/ post-test assessment), group (cognitive training/no cognitive training) and interval (7-day/14-day)

Training groups	7-day interval		14-day interval	
	*Pre-test M* (*SD*)	*Post-test M* (*SD*)	*Pre-test M* (*SD*)	*Post-test M* (*SD*)
**Task-switching task**				
*RT in ms*				
Switching costs	215 (214)	145 (175)	243 (287)	190 (139)
Mixing costs	906 (283)	695 (208)	960 (441)	780 (314)
*Accuracy rate in %*				
Switching costs	1.83 (3.81)	1.06 (3.54)	4.90 (7.06)	2.15 (3.73)
Mixing costs	-1.06 (6.69)	1.00 (1.94)	1.35 (4.80)	.45 (3.91)
**Far transfer tasks**				
*Updating*				
Total RT in ms	1112 (170)	1080 (136)	1108 (190)	1160 (178)
Accuracy in %	71.45 (11.63)	73.66 (11.81)	64.65 (19.00)	66.10 (12.90)
*Inhibition*				
Stroop interference	13.04 (5.50)	12.09 (5.56)	16.69 (7.99)	14.37 (7.03)
*Prospective memory*				
Accuracy in %	.67 (.48)	.89 (.32)	.40 (.50)	.80 (.41)
Questionnaire	3.70 (.71)	3.66 (.79)	3.97 (.44)	3.71 (.67)
**Control groups**	**7-day interval**		**14-day interval**	
**Task-switching task**				
*RT in ms*				
Switching costs	236 (287)	236 (332)	351 (283)	269 (287)
Mixing costs	794 (467)	898 (487)	1159 (489)	903 (464)
*Accuracy rate in %*				
Switching costs	1.71 (5.51)	4.81 (5.27)	3.17 (3.90)	3.66 (4.25)
Mixing costs	1.95 (7.28)	1.62 (3.98)	.72 (4.02)	.86 (5.74)
**Far transfer tasks**				
*Updating*				
Total RT in ms	1146 (108)	1093 (137)	1086 (182)	1041 (136)
Accuracy in %	62.72 (11.33)	66.13 (12.40)	61.58 (19.32)	66.91 (18.94)
*Inhibition*				
Stroop interference	16.50 (5.32)	16.80 (7.00)	19.36 (8.82)	15.55 (5.61)
*Prospective memory*				
Accuracy in %	.57 (.51)	.71 (.46)	.55 (.51)	.69 (.47)
Questionnaire	3.91 (.63)	4.10 (.52)	3.70 (.90)	3.75 (.76)

For the *inhibition* task, analyses revealed a significant main effect for time (*F*(1, 84) = 7.16, *p* < 0.01, η^*2*^_*p*_ = 0.08), indicating reductions in stroop interference from pre- to post-assessment for all participants. However, the time × group interaction did not reached significance, *F*(1, 84) = 0.01, *p* = 0.92, η^*2*^_*p*_ = 0.00. For the *updating* domain, a significant main effect for time but no time × group interaction was found for overall correct responses, *F*(1, 77) = 4.20, *p* < 0.05, η^*2*^_*p*_ = 0.05 and *F*(1, 77) = 0.40, *p* = 0.53, η^*2*^_*p*_ = 0.01, respectively.

In regard to *prospective memory* performance, no significant time × group interaction effect was found for the lab-based measure, *F*(1, 84) = 2.18, *p* = 0.14, η^*2*^_*p*_ = 0.025. However, for the self-report measure, there was a trend towards a time × group interaction effect (i.e., transfer effect), *F*(1, 64)^8^ = 3.49, *p* = 0.066, η^*2*^_*p*_ = 0.05. Participants training task-switching reported less problems with prospective memory after training than participants in the control groups.

#### Additional Analyses

Exploring possible moderators for performance gains during training, a significant moderate correlation for well-being and training gains in switching costs RT was revealed (*r* =  − 0.36, *p* < 0.05), indicating that participants who reported lower well-being showed smaller training gains in total reaction time from first to last training session. In addition, accuracy switching costs at T1 correlated significantly with training gains in switching costs RT (*r* = 0.34, *p* < 0.05). There was no other significant correlation between training gains (RT and accuracy) and the investigated variables (time between assessments, number of performed training sessions, age, education, weekly working hours, computer competence, emotional exhaustion, T1 switching costs (RT), importance of cognitive fitness for work) (see Table 6 in the Appendix).

## Discussion

Even healthy adults have to face age-related decline across several cognitive domains that partly starts as early as in their late twenties (Park & Shaw, 1992; Park et al., 1996; Salthouse, 1996; Salthouse & Babcock, 1991). Correlational data supporting a disuse hypothesis of cognitive aging suggest that individuals engaging in intellectual and social activities during adulthood undergo less age-related cognitive declines (e.g., Amieva et al., 2010; Ihle et al., 2018, 2020, 2020; Ihle, Bavelier, et al., 2020; Stern, 2009; Wilson et al., 2013). Consequently, to promote quality of living and independence in old age, the aim of cognitive aging research not only consists in identifying interventions that help to promote or maintain cognition in the process of aging, but it also has started to investigate feasibility and applicability of established laboratory interventions in everyday life. Thus, cognitive training programs have to answer two questions: First, whether these programs are able to enhance or maintain cognitive functioning that ideally transfers to everyday life activities (*effectiveness*), and second, what conditions lead to a successful implementation of the training programs in everyday life of the target group (*feasibility*). The current study aimed at investigating later question by implementing an executive control training (here: task-switching training) into everyday life of a working population and exploring four areas relevant for feasibility: adherence, effectiveness, efficiency, and generalization. Eighty-one middle-aged caregivers were instructed to regularly perform a self-administered task-switching task at home. To our knowledge, the present study was the first examining feasibility of a task-switching training in everyday life of employees (caregivers). We first discuss the results together with their limitations to answer the four feasibility questions before pointing out conceptual, methodological, and practical implications.

### Adherence to a Home-Based Task-Switching Training in Middle-Aged Caregivers

Regarding the integration of an executive control training into everyday life with low experimental control, our results suggest that performing a task-switching training regularly at home is, in principle, possible for middle-aged caregivers. However, as a limitation, only a small number of caregivers practiced the training task exactly the number of times instructed. Roughly one quarter of the participants decided not to practice any session at all (0 sessions). Furthermore, most of the participants conducted less than the instructed training sessions, whereas others trained even more than the recommended number of sessions.

Thus, the present results are contrary to classical laboratory studies (e.g., Karbach & Kray, 2009; Kray & Fehér, 2017; Zinke et al., 2012) in which feasibility is not a research questions, the number of training sessions is rather constant (± 1 at most), and drop-out or non-performing rates are comparatively low. However, evidence for lower adherence rates and high drop-out rate in real-life is provided by training studies conducted outside the laboratory. For example, Wolinsky et al. (2013) reported that 28.3% of their middle- and old-aged adults (> 50 years) who trained at home performed less than 1 h (out of 10 h) of training in a visual speed task. In a follow-up study, Wolinsky et al. (2020) found also a great variety of adherence to a visual processing speed training a community-based sample of older adults (> 55 years). Chan and colleagues (2016) conducted a field training study targeting older adults (60–90 years) in a community setting which included homework as well as group training for an extensive time (> 150 h). Here, also a high drop-out rate (7 out of 25) was reported. In these studies, about one quarter of the participants dropped-out or decided not to perform the training (see also Hardy et al., 2015; Ng et al., 2020). These results are congruent to the findings in 4.

Interestingly, the reported adherence rate is also comparable to compliance rates in medical health studies. For instance, DiMatteo (2004) found in her meta-analysis (*k* = 569) an average non-adherence rate of 24.8%. Moreover, adherence is lower for more complex regular health behavior (e.g., diet 59.3%, appointments 65.9%, vs. taking medication 79.4%) (see also Miller & DiMatteo, 2015). As main reasons for these low compliance rates, two factors can be discussed: *cognition* and *motivation*. Research showed that remembering to implement intended actions in the future (PM) is one of the most challenging cognitive tasks (e.g., Crovitz & Daniel, 1984; Haas et al., 2020; Maylor, 1990). This is particularly true for individuals with an instable lifestyle without daily routine (e.g., Park et al., 1999; Schnitzspahn et al., 2011; Stine-Morrow et al., 2008). Correspondingly, this may be true for the present sample as caregivers usually work irregular hours (shiftwork). Consequently, their everyday life is unstable and not marked by daily routine. In line with studies providing evidence for lower cognitive performance in shift-workers (e.g., Jørgensen et al., 2020; Marquie et al., 2015), PM performance might be diminished in the current sample. Taken together with the instable lifestyle, this could be one reason for the low adherence rate found in this study. Strategies supporting the linkage of regular intended behavior (e.g., perform training) to stable contextual cues (such as the breakfast table) have proven to be very successful in enhancing PM performance (e.g., implementation intentions, Gollwitzer, 1999). More practical, the usage of external reminder (e.g., messages, e-mail) or synchronization of training and working schedule could also lead to higher training adherence rates in middle-aged nurses. Thus, incorporating these strategies in order to support the performance of regular intended behavior (e.g., cognitive training) might be a good aid for care workers. Further studies are needed to confirm this assumption and investigate possible mechanism to support shift workers effectively. In addition, shift work and high work demands, typically associated with nursing, increases the likelihood of emotional exhaustion (e.g., Brom et al., 2015), which in turn may decrease motivation and/or the ability to find time to perform cognitive training. Accordingly, we found that participants who did not perform any training session at home reported a higher score in emotional exhaustion at the beginning of the study.

Several theories on behavioral change propose moderators and mediators for the execution of the behavior (e.g., the transtheoretical stages of change model, Prochaska & DiClemente, 1983; Rubicon model of action phases, Heckhausen & Heckhausen, 2010). Motivation and consciousness about the necessity of the behavioral change are important factors. In this line, Harrell et al. (2019) showed that the willingness to engage in cognitive training depends on the individual perception of cognitive training efficacy as well as participants’ self-perceived cognitive deficit (in study 1). It can be assumed that the urge to change routine behavior and implement cognitive training in everyday life might be less pronounced in 4 as in health behavior studies in which the omission of behavior (i.e., taking medication or performing tests) can have immediate severe consequences. Moreover, particularly laboratory studies booster motivation by giving (instant) external gratification (e.g., money, vouchers, appreciation by the researcher during training) (see, e.g., Brom et al., 2014; Karbach & Kray, 2009; Zinke et al., 2012). Besides the performance feedback at the end of each block during the training, the current study does not give any external gratification for the task-switching training per se and thus relies more on internal motivation. As the training follows a prevention approach, the low compliance rate, which is even lower than in medical adherence studies, is not too astonishing.

To aim at the motivational issues concerning home-based cognitive training, different studies suggest to design training programs that are tailored to the specific needs of the target group (see, e.g., Smid et al., 2020). Deveau and colleagues (2014) propose integrating principles of perceptual learning (PL) and computer science into the training design. However, studies on increased gamification do lead to inconsistent results (e.g., Dörrenbächer et al., 2014; Johann & Karbach, 2020; Katz et al., 2014. Some other authors used paper pencil training task for older adults which might be more accessible for this middle-aged workers (see, e.g., Gajewski et al., 2017). Thus, designers of further cognitive training programs have to balance the two aims: (1) targeting effective relevant cognitive processes and (2) creating a motivating program surface for the target group. Adaptive or multidomain cognitive trainings that include external gratification (e.g., vouchers when reaching a certain accuracy rate or level, competition) might target the motivational aspect while still aiming at the relevant cognitive processes.

Next to cognition and motivation, many other variables can also be put forward as possible explanation for the low adherence rates or the reason why some participants neglected to train (e.g., personality, self-control, self-efficacy, see, e.g., Brom et al., 2014). Future feasibility studies might consider personal differences of non-responders in their study design.

### Effectiveness, Efficiency, and Generalization of a Home-Based Task-Switching Training

From previous research (see above), it can be argued that diminished adherence is an unavoidable part of field research. Thus, as the central aim of the present study was to investigate implementation of a laboratory training regime in everyday life, we conducted further analyses to explore the feasibility questions regarding effectiveness, efficiency, and generalization. We believe, the present results (with all its limitation from a laboratory perspective) can point towards important implications for further cognitive research.

For the home-based task-switching training, the present study found a significant decrease in overall RT and RT switching costs as well as an increase of accuracy rate between the first and fourth and last training session, respectively. This suggests a successful improvement in the training as such and may point towards the effectiveness of the training itself, even when it was applied self-administered at home. The observed training gains between the first and the fourth training sessions are consistent with previous laboratory studies using a similar task-switching training paradigm as well as a similar numbers of training sessions (3–5) (e.g., Brom & Kliegel, 2014; Karbach & Kray, 2009; Kray & Fehér, 2017; Zinke et al., 2012). These studies predominantly revealed improvements in RT measures. Consistently, large RT improvements were also observed in the training of other executive function tasks, such as measuring inhibitory control (e.g., Enge et al., 2014).

Interestingly, our results revealed a similar magnitude of training gains between first and fourth training sessions and first and last training sessions. At the first sight, this result is in line with the meta-analytic results who found that training gains did not differ according to total training time (e.g., Karbach & Verhaeghen, 2014; Mewborn et al., 2017). However, looking at the current results more closely, there is a difference in the effects of the two interval groups. Between the first and the fourth training sessions, the training groups did not differ with regard to their training gains. Yet, a comparison of RTs between first and last training sessions showed a stronger decrease for the 7-day interval group, as the RT for switching and for non-switching trials was reduced by almost 50%. Moreover, training gains between first and last training sessions in RT switching costs were also numerically larger for the 7-day interval group than for the 14-day interval group. These results might suggest that over a longer course of training (> 4 sessions), the 7-day interval group seemed to profit most from performing the task-switching training. This is in line with previous studies which applied weekly training sessions (e.g., Karbach & Kray, 2009; Kray & Fehér, 2017; Zinke et al., 2012). Furthermore, some meta-analytic results suggest that 1–3 training sessions per week have the greatest effect (e.g., Kelly et al., 2014). However, so far, most training studies investigated weekly or even daily training, implementation of the later regime might be very challenging or even unrealistic in everyday life of a working population. Thus, a weekly training interval might be most practical to conduct task-switching training or cognitive training in everyday life. To our knowledge, none of the previous training studies compared weekly and fortnightly training.

Regarding the transfer to non-trained tasks, we only found effects for the (non-trained, novel) task-switching task (near transfer) but not for the inhibition, updating or PM laboratory measures (mid and far transfer). The significant pre-post-test reductions in accuracy switching costs and the trend in RT mixing costs in comparison to the control groups point towards the fact that improved task-switching abilities in the training groups were acquired due to training. Again, there was a trend of greater improvement (RT mixing costs) for the 7-day interval group. Using a task-switching task with a different paradigm, our results are in line with the previous lab studies (e.g., Brom & Kliegel, 2014; Dörrenbächer et al., 2019; Karbach & Kray, 2009; Karbach et al., 2010; Kray & Fehér, 2017; Zinke et al., 2012). However, with small- and medium-sized effects, the near transfer effects were comparatively smaller in the current study (despite having obtained comparable effect sizes in the training task itself). For the inhibition and updating task, the study revealed only time effects; transfer effects were not detected. Our findings are in line with a study by Kray and Fehér (2017), but contrary to Karbach and Kray (2009) and Zinke et al. (2012), who provided evidence for transfer to other executive control measures. However, the results are mixed and the effect sizes for far transfer are generally very small (see e.g., Karbach & Verhaeghen, 2014). In addition, some meta-analytic reviews found trainings performed at home to be less effective than more controlled forms of training (see Kelly et al., 2014; Lampit et al., 2014). Thus, the absence of mid and far transfer in the current study could be due to the general small effects of far transfer or the uncontrolled implementation environment. Hence, a generalization of laboratory results in regard to mid and far transfer can neither be supported nor ruled out by the current study results. Thus, further studies that apply the current training regime both in the laboratory and at home are needed to corroborate the current finding.

The rationale behind investigating transfer of task-switching training to PM as a proxy for everyday relevant cognitive performance lies in the strong empirical evidence for the relationship between task-switching and PM performance (e.g., Azzopardi et al., 2017; Salthouse et al., 2004; Schnitzspahn et al., 2013; West et al., 2011). Our results suggest that the training-induced improvements in task-switching do not lead to improved PM performance, at least not in the laboratory. Using a more complex PM measure (blood pressure monitoring), Brom and Kliegel (2014) also found no transfer of five sessions of task-switching training to PM performance in older adults. However, contrary to the results in the laboratory task, participants in the training groups reported a decrease in subjective PM failures, which suggests that there may be in fact some potential to real-life outcomes (see Brehmer et al., 2012, for similar results on self-reported cognitive failures of a lab-based WM training). Of course, stronger evidence is needed and further research will be necessary to investigate this question. Nevertheless, this result could indicate that executive control trainings do transfer to everyday memory performance and standard laboratory tasks might be limited in representing everyday life tasks or cognitive challenges. Thus, our results hint towards including more relevant transfer measures characterized by high ecological validity in cognitive training studies.

Lastly, when interpreting the results in regard to effectiveness and generalization, two methodological limitations need to be considered. First, from an experimental psychology perspective, the use of an active control group should lead to a different outcome in regard to the effectiveness of the task-switching training than the used passive control group (e.g., Melby-Lervåg & Hulme, 2016). Unfortunately, including an additional active control group was not possible in the current study. However, Au et al. (2016) could not find “evidence that control group type meaningfully influences effect sizes from objective cognitive measures” (p. 208) in their recent extensive meta-analytic review. Furthermore, the authors concluded that studies with passive control groups should not be considered less valid than studies with active control groups. Nevertheless, until the debate concerning the use of control group type is resolved, the use of both passive and well-designed active control groups should be recommended where possible in further studies. Second, in regard to task-switching training, we did not evaluate maintenance of effects. Due to the extended study period, a follow-up test session was not practicable. However, to answer the feasibility and efficiency questions for the first time, one post-test should suffice. Moreover, as we did not find any effect for mid and far transfer, a follow-up assessment is not expected to lead to different results. Nevertheless, future studies might include a follow-up session were possible.

Taken together, in line with previous training and medical compliance research, *adherence* is a challenge for studies conducted in everyday life requiring self-initiated execution of regular behavior; this applies to the current training study as well. Nevertheless, task-switching training conduced self-directed at home by compliant caregivers (> 50% of the sessions) was only *effective* with regard to improving task-switching abilities and may even transfer to everyday life. In regard to *efficiency*, employees practicing in a 7-day interval (weekly) seem to benefit more from applying the training than caregivers training only every second week. As the results for mid and far transfer are contrary to some findings in previous laboratory studies, there is only limited evidence in regard to *generalization* of laboratory results to everyday life. In conclusion, the current results suggest that the implementation of a self-administered task-switching training at home is possible for middle-aged caregivers. However, as effectiveness was limited to training and near transfer, more studies are needed to investigate feasibility of laboratory training regimes in everyday life of working adults. Further research should aim at exploring strategies and programs that facilitate the performance of required behavior and increase the motivation potential as well as the accessibility of training programs substantially.

## Conclusion and Applied Implication

From a social and applied perspective, one of the central questions is how can age-related cognitive decline—relevant for everyday life performance—be prevented or reduced for the normal aging population in an economical way? The current study aimed exactly at this question in taking an established laboratory training task and applying it in everyday life of a working population. In line with previous aging studies (e.g., Kelly et al., 2014; Lampit et al., 2014), we found that home-based training was less effective than training in laboratory studies using a similar training paradigm. In addition, compliance rate was relatively low. Only a small number of care workers that was instructed to implement training sessions at home did perform the sessions exactly as instructed. Research suggests that in general, transfer of laboratory results into everyday life is challenging (e.g., Vanhove & Harms, 2015). Three points can be proposed as primary reasons therefore: high *experimenter control*, *manipulation of motivation*, and *preselected sample* in laboratory studies. First, due to the social interaction, experimenter control in the laboratory can lead to non-specific effects that are not related to the experiment itself (e.g., Hawthorne effect, see McCambridge et al., 2014; Stereotype threat, see Lamont et al., 2015). Further, in laboratory studies, participants are guided through the experimental procedure by the experimenter; thus, participants’ own initiative to remember to train (such as in our study) does not play a critical role. Second, most laboratory studies apply external rewards (e.g., money, vouchers), which are not available in everyday life. Gratifications might influence the effectiveness of a training in the laboratory and lead to an underestimation of the adherence problem in everyday life. Third, individuals participating in laboratory studies normally represent a selected sample. For example, older adults answering an advertisement for a training study might be more motivated to change their behavior than older adults not responding. In addition, many laboratory training studies are conducted with students or individuals with a higher educational background. Taken together, different mechanism can be proposed for the laboratory-field gap. To answer questions about basic underlying processes or principles of cause and effect, highly controlled laboratory experiments are vital. However, adherence and performance rates found in 4 might represent compliance as well as performance of middle-aged working adults more closely than laboratory studies. Thus, we want to encourage laboratory training studies to integrate more ecological valid measures and representative populations to become more relevant for everyday life and society.

In addition, taken together with adherence research results, the current study underlines the fact that not every individual is able or willing to perform a regular behavior that can maintain health or prevent health decline. Nevertheless, to account for the trends in society (e.g., aging population and workforce) and the costs of nonadherence, health research needs to identify factors that hinder performance of regular health-related behavior and to find ways to motivate specific target groups to higher adherence rates. Specifically, in the current study, emotional exhaustion/well-being and the in-between sessions’ interval seem to influence adherence and training gains, respectively.

## Data Availability

The datasets generated and analyzed during the current study are not publicly available due to the privacy arrangements with the participants as well as the participating organizations but are available from the corresponding author on reasonable request.

## Footnotes

1 For data analyzes, practice blocks and first trials in each block as well as trials with reaction times faster than 100 ms were excluded.

2 Due to voluntariness, not all study participants completed the questionnaires at pre- and post-assessment.

3 Number of sessions as recorded by the program.

4 Excluding these two participants from the analyses revealed similar results as the ones based on the full sample.

5 The decisions was based on following considerations: First, participants did not significantly differ in regard to age, gender, average working hours and pre-test task switching performance from participants not instructed to train (control group). Second, result pattern stayed the same when adding these participants to the control groups.

6 Individual number of training sessions was added as co-variable. Number of training sessions did not correlated significantly with training gains (*r* = .01—.22, *p* = .18—.94).

7 When adding RT mixing costs (T1) as control variable, results regarding GroupxTime interactions for transfer measures did not change. However, the trend in the prospective memory self-report measure became non-significant (*p* = .17).

8 Complete pre- post-assessment data missing for 20 participants (5 training group, 15 control group).

## Declarations

### Appendix

Table 4

**Table 4:** Participants’ mean scores and standard deviations on demographic, health, and cognitive variables shown separately for study sample and excluded participants

	Study sample	Excluded participants	*t*-/ *χ*^*2*^-value
N	99	17	
Interval (7-day)	42	8	.13^a^
Time between assessments	14.69 (4.79)	15.62 (4.86)	.74^b^
*Demographics*			
Sex (female)	96	17	.53^a^
Age (years)	52.59 (5.18)	53.18 (3.86)	.55^b^
Education (years)	9.89 (1.02)	9.67 (.78)	− .72^b^
Weekly working hours	31.62 (6.07)	31.46 (8.14)	− .09^b^
Tenure	14.21 (9.79)	15.63 (10.04)	.39
Computer competence (self-report)	5.16 (2.39)	5.19 (2.42)	.04^b^
*Health*			
Emotional exhaustion	3.25 (1.48)	4.31 (1.63)	2.48^b^*
Well-being/depression	2.79 (1.11)	2.29 (1.26)	− 1.57^b^
*Cognitive variables*			
Switching costs (RT)	261 (270)	206 (282)	− .73^b^
Mixing costs (RT)	974 (440)	1045 (590)	.56^b^
Switching costs (%)	2.93 (5.02)	3.67 (6.00)	.52^b^
Mixing costs (%)	.78 (5.39)	3.27 (6.97)	1.60^b^

*Note*. *N* = 101–116

^*^*p* < .05

^a^*χ*^*2*^-value

^b^*t*-value

Table 5

**Table 5:** Participants’ mean scores and standard deviations on demographics, health, cognitive, and training-related variables as a function of training intensity

	Trainings group (> 4 sessions)	Trained < 50% (1–3 sessions)	Rejected (0 sessions)		Non-contact group	*F*-/*χ*^*2*^-value
N	38	11	18		32	
Interval (7-day)	18	3	6		15	2.28^a^
Time between assessments (in weeks)	14.62 (4.79)	15.77 (5.96)	15.44 (5.02)		13.97 (4.30)	77.55*
*Demographics*						
Sex (female)	38	11	17		30	3.05^a^
Age (years)	52.55 (5.46)	51.10 (4.72)	51.72 (6.09)		53.59 (4.41)	.84^b^
Education (years)	10.11 (.83)	9.40 (.97)	10.00 (1.31)		9.72 (1.03)	1.71^b^
Tenure	15.40 (8.39)	15.25 (15.66)	14.29 (8.41)		12.97 (9.94)	.36
Weekly working hours	32.13 (4.30)	29.70 (9.88)	31.38 (7.45)		31.80 (5.66)	.42^b^
Computer competence (self-report)	5.16 (2.31)	6.36 (.92)	5.11 (2.78)		4.77 (2.56)	1.20^b^
*Health*						
Emotional exhaustion	3.05 (1.23)	2.54 (1.11)	4.05 (1.77)		3.26 (1.57)	2.77^b^*
Well-being	2.84 (.97)	3.14 (.95)	2.62 (1.22)		2.72 (1.25)	.52^b^
*Cognitive variables*						
Switching costs (RT)	230 (252)	179 (236)	350 (240)		276 (311)	1.20^b^
Mixing costs (RT)	934 (371)	967 (328)	1136 (449)		932 (533)	1.00^b^
Switching costs (%)	3.45 (5.88)	2.82 (3.28)	2.67 (3.65)		2.50 (5.19)	.23^b^
Mixing costs (%)	.21 (5.82)	.64 (2.06)	.61 (3.43)		1.59 (6.52)	.39^b^
*Training variables*						
Enjoyment of training	6.95 (1.91)	6.36 (2.29)	-	-	- -	n.s
Rated importance of cognitive fitness for work	8.78 (1.38)	8.91 (1.14)	8.17 (1.46)		Not measured	1.50^b^

*Note*. **p* < .05

^a^*χ*^*2*^-value

^b^*F*-value

*N* = 89–99

Table 6

**Table 6:** Correlation between training gains and possible moderator variables

	1	2	3	4	5	6	7	8	9	10	11	12	13
*Training gains*													
1. Switching costs (RT)	1												
2. Switching costs (error)	.072	1											
*Possible influencing variables*													
3. Time between assessments (in weeks)	.239	.008	1										
4. Number of performed training sessions	.013	− .190	− .032	1									
5. Age	− .019	− .135	.040	.132	1								
6. Education (years)	− .259	− .092	.012	.049	− .030	1							
7. Weekly working hours	− .025	− .045	− .015	.087	− .075	− .019	1						
8. Computer competence (self-report)	.083	.192	− .184	.008	− .129	.220	− .089	1					
9. Emotional exhaustion	.187	− .057	.034	− .188	− .112	.115	− .136	.131	1				
10. Well-being	− **.362**^*****^	.274	− .091	.094	.087	.041	− .108	− .068	− .587^**^	1			
11. T1 Switching costs (RT)	.173	.111	.146	− .174	.156	− .012	− .017	.019	.074	− .090	1		
12. T1 Switching costs (%)	**.337**^*****^	.244	.249^*^	.259^*^	− .095	− .108	.267^*^	− .120	− .178	− .038	.066	1	
13. Rated importance of cognitive fitness for work	− .194	− .149	− .065	− .042	− .396^*^	.176	− .005	− .053	− .062	− .198	− .181	− .115	1

Note. **p* < .05

^**^*p* < .01

^***^*p* < .001

*N* = 35–38. Training gains = last training – first training
